# A Low Complexity Rolling Bearing Diagnosis Technique Based on Machine Learning and Smart Preprocessing

**DOI:** 10.3390/s23177546

**Published:** 2023-08-30

**Authors:** Ada Fort, Elia Landi, Marco Mugnaini, Valerio Vignoli

**Affiliations:** Department of Information Engineering and Mathematics, University of Siena, 53100 Siena, Italy; elia.landi@unisi.it (E.L.); marco.mugnaini@unisi.it (M.M.); valerio.vignoli@unisi.it (V.V.)

**Keywords:** fault bearing fault diagnosis, embedded systems, condition monitoring, machine learning

## Abstract

In this work, we present a diagnosis system for rolling bearings that leverages simultaneous measurements of vibrations and machine rotation speed. Our approach combines the robustness of simple time domain methods for fault detection with the potential of machine learning techniques for fault location. This research is based on a neural network classifier, which exploits a simple and novel preprocessing algorithm specifically designed for minimizing the dependency of the classifier performance on the machine working conditions, on the bearing model and on the acquisition system set-up. The overall diagnosis system is based on light algorithms with reduced complexity and hardware resource demand and is designed to be deployed in embedded electronics. The fault diagnosis system was trained using emulated data, exploiting an ad-hoc test bench thus avoiding the problem of generating enough data, achieving an overall classifier accuracy larger than 98%. Its noteworthy ability to generalize was proven by using data emulating different working conditions and acquisition set-ups and noise levels, obtaining in all the cases accuracies greater than 97%, thereby proving in this way that the proposed system can be applied in a wide spectrum of different applications. Finally, real data from an on-line database containing vibration signals obtained in a completely different scenario are used to demonstrate the distinctive capability of the proposed system to generalize.

## 1. Introduction

In industrial systems, rotating machines play a vital role. Rolling element bearings are often one of the most vulnerable components in machinery. Their health condition significantly impacts the machine performance, efficiency, stability, and life cycle [1], moreover bearing fault is one of the most common causes for machine catastrophic failures. As an example, some studies [2,3] reveal that bearing faults are the most common cause of induction engine failures, being responsible for one-third of all defects. In this context, several types of failures are possible in bearings, due to mechanical fatigue, ambient contaminations, and bearing currents [4]. Furthermore, in the context of renewable energies, such as in wind turbine applications, the reliability of the bearings is of utmost importance. Here, issues such as plastic deformation, wear, cracks, fractures arising also from insufficient lubrication or contamination can lead to failures in bearing components [5,6]. In nuclear power plants and gas turbines, bearings are critical components whose health is directly linked to the safety of the entire plant [7,8,9]. As a result, condition monitoring and fault diagnosis of rolling bearings has become an essential area of development and engineering research. In this context, measuring mechanical vibration signals is one of the most important means for understanding processes related to bearing faults, as they in principle can be used to detect and recognize various types of faults [1,10]. To detect faults, vibration sensors (accelerometers) are mounted on the machine, as close as possible to (or on) the bearings, providing continuous monitoring of the machinery vibrations, which are acquired and processed on-line. The success of fault detection methods relies mostly on the quality of the collected vibration signals but can be also significantly enhanced by the selection of appropriate signal processing and feature extraction techniques [11]. Actually, the quality of the vibration signal is often not considered with the necessary care. Vibrations signals from rolling bearings are in fact wideband signals, with high frequency components, but encompassing low-frequency significant characteristics. To preserve the information content of these signals, and to provide a high probability of incipient fault detection from their analysis, careful positioning of the sensors is required together with the use of accurate and wideband sensors, conditioning electronics, and acquisition systems. As far as the fault location is concerned the problem becomes even harder. In fact, in this case, besides using a high-quality measurement and monitoring system the behavior of the bearing must also be close to the ideal one, when rolling elements are not subject to sliding. Moreover, the vibration signal shape depends on the machine rotation speed.

When one of the elements of the bearing is damaged, its defect impacts on the other components during the rotation and each impact starts a transient vibration, whose intensity is determined by the severity of the fault. As such, vibration signals caused by faults can be seen as trains of pulses repeated with a periodicity dictated by the rotation speed of the damaged components.

The time-domain analysis is the most direct approach for detecting faults in bearings since it is based on the evaluation of scalar indices to measure the energy and to spot the presence of pulses [1]. This analysis usually aims at evaluating different scalar parameters to highlight the presence of any type of fault, among the most used parameters there are the peak value, peak-to-peak value, the root-mean-square (RMS), and the crest factor, skewness, kurtosis, and spectral kurtosis, impulse factor, shape factor, and clearance factor.

On the other hand, frequency-domain or spectral analysis is the most employed method for fault recognition and location. This method exploits the transformation of the vibration signals into the frequency domain using discrete Fourier transform (DFT), allowing thus for detecting the characteristic periodicity (fundamental frequency) of each type of fault.

Actually, real fault vibration signals are complex and can be the combination of periodic components and non-stationary ones, and this behavior suggests the application of time-frequency analysis. Many time-frequency techniques have been applied for bearing diagnosis, such as short time Fourier transform (STFT), Wigner-Ville distribution, and wavelet transform.

There are some well-known drawbacks of the above-mentioned traditional methods: time domain methods are not suitable for fault locations, frequency domain methods are often disturbed by noise or temporary non stationarity due to sliding, whereas time-frequency approaches suffer from the selection of the right time window length which changes with respect to the operating conditions, or for Wavelet from selecting the appropriate mother wavelet and decomposition level.

As a result, many researchers have sought in machine learning (ML) techniques solutions able to overcome some of these problems. During the last decades a huge quantity of research work on this topic has led to a variety of different possible solutions, such as artificial neural networks (ANN), support vector machines (SVM), neural fuzzy network [12,13] Bayesian networks [14,15], self-organizing maps [16], extreme learning machines (ELM) [17,18], linear discriminant analysis [19,20], independent component analysis [21], softmax classifiers [1,22], manifold learning [23,24], and canonical variate analysis [25,26]. Since traditional ML need, in any case, a great effort in extracting appropriate features from the raw signals, more recently, deep learning (DL) based methods, including convolutional neural network (CNN), auto-encoder (AE), deep belief network (DBN), recurrent neural network (RNN), and generative adversarial network (GAN) have been proposed [1,26]. In many cases authors claimed to cope with noisy environments and variable working conditions [27,28]. Most of the literature applying these ML algorithms report satisfactory results with classification accuracy over 90%.

The main drawback in all these approaches is the need of a very large quantity of labelled data to train and properly verify the diagnosis system, and this is especially true for DL techniques. In fact, when using small datasets, classical ML algorithms can compete with or even outperform deep learning networks [29]. The needed dataset should indeed represent the behavior of the different faults in all the possible operating conditions. This, in principle, is viable resorting to experimental data, but injecting controlled faults and measuring vibration signals experimentally is really expensive and time consuming. Therefore, many of these works propose solutions developed for exploiting the very limited number of examples present in the public online databases [26]. Therefore, their capability of generalizing is often not proven.

Additionally, often experimental signals stored in these databases (including the Case Western Reserve University, CWRU) obtained with simple artificially injected defects, and labelled as examples of single fault signatures, contain portions affected by sliding, or non-stationarity of the machine rotation regime or of the faulty condition, which are not isolated and labelled as such. These signal parts should be discarded or isolated when training the diagnosis system, reducing further and dramatically the size of the exploitable datasets.

In this work we proposed a diagnosis system for rolling bearings, based on the simultaneous measurement of vibration and machine rotation speed, that combines the robustness of simple time domain methods for fault detection, with the potential of ML methods for fault location. The fault classifier leverages a simple preprocessing algorithm that allows for minimizing the dependency of the classifier performance on the machine working conditions, on the bearing model, and on the acquisition system set-up. The overall diagnosis system is based on light algorithms with reduced complexity and hardware resource demands and is designed to be deployed in embedded electronics. The fault diagnosis system is trained using emulated data, exploiting an ad-hoc test bench thus avoiding the problem of generating enough data.

The novelty of the proposed approach resides mainly in the tailored preprocessing algorithm, which leverages the transformation of a time signal into a gray scale image, thus permitting the usage of many available neural models for the following classification task. But beyond this benefit, which has already been exploited in many different works [30,31,32], the designed preprocessing produces a far more crucial advantage: it allows for cancelling the dependence of the signal on the rotation speed of the machine and on the sampling time. Moreover, this simple and fixed preprocessing step allows for extracting the peculiar structure of the vibration signal related to different faults, which is independent also from the mechanical component, sensor, and front-end responses. Due to this characteristic, the proposed technique allows for overcoming many of the problems of similar ML based methods. In fact, the diagnosis system can be trained with emulated data, which are available in large quantities, and allows for avoiding cumbersome and machine-dependent experimental procedures. Finally, a trained network serves for the condition monitoring of a given model of roller bearing mounted on any machine and rotating at any speed, with no need of adaptation.

The approach presented in this paper holds significant promise as it capitalizes on the growing availability of low-cost accelerometers with extended bandwidth capabilities of up to 20 kHz. These accelerometers, developed using state-of-the-art Micro-Electro-Mechanical Systems (MEMS) technologies, accompanied by embedded processing, offer a tangible opportunity to deploy widespread distributed low-cost diagnosis systems.

## 2. Bearing Failures: Background and Description

**Figure 1:**
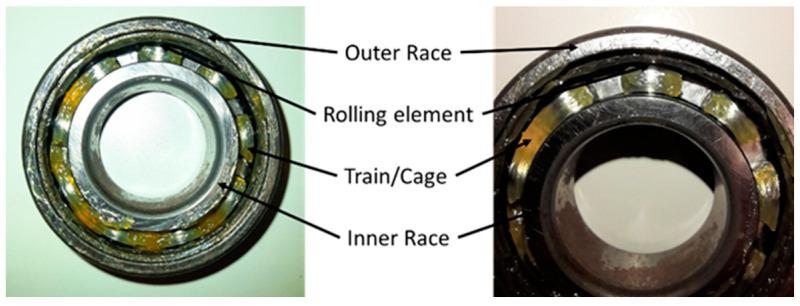
Bearing structure and components. The main components where wear typically occur are the inner and the outer race as well as the rolling elements.

A rolling bearing comprises three main components: the outer race, the inner race, and the rolling elements (or ball), as shown in Figure 1.

Wear typically damages one of these three components. In the event of a defect in the bearing components, vibrations are generated. The magnitude of these vibrations is directly linked to the severity of the damage. Moreover, the specific damaged bearing element, the type of bearing, and the rotational speed of the machine all contribute to determining the frequency content of the vibration signal.

### 2.1. Mathematical Description of Bearing Single Failures

**Figure 2:**
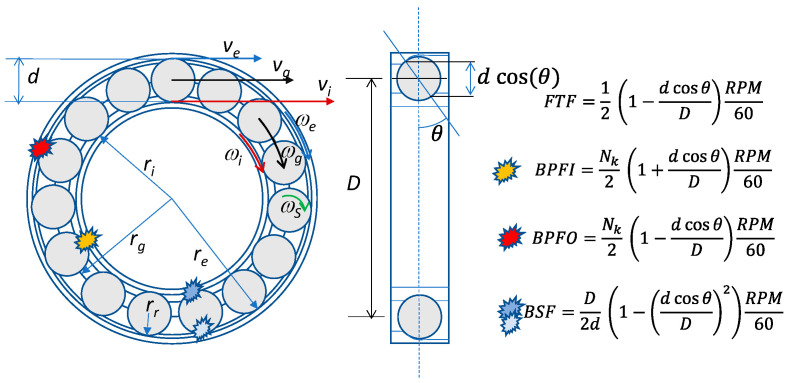
Roller bearing geometry, representation of the defects placement and characteristic frequencies related to them. Wear occurs in components that mechanically interact during operation: the outer race with rolling elements, and the inner race with rolling elements. FTF is the train/cage frequency, BPFI is the inner race failure to rolling element impact frequency, BPFO is the outer race failure to rolling element impact frequency, and BSF is the rolling element failure to two-race impact frequency.

When one of the elements of the bearing is damaged, its defect periodically impacts on the other components during the rotation. Each impact excites the mechanical resonance of the metallic structure near the bearing, whose fundamental frequency is usually much higher than the frequencies of the impacts, resulting in a transient vibration that can be described by a decaying sine wave. Therefore, the overall vibration signal caused by a fault, in the absence of sliding of the rolling elements (pure rolling), is a train of transient vibrations repeated with a fundamental frequency dictated by the rotation speed of the faulty component. In Figure 2 the geometry of a roller bearing is represented, together with the characteristic fundamental frequencies of the vibration signals generated by the different component faults. In particular, the ball pass frequency on the inner race (BPFI), is the fundamental frequency for defects of the inner race, the ball pass frequency on the outer race (BPFO) is the one for faults of the outer race, whereas the ball spin frequency (BSF) characterizes faults of the balls (or rolling elements) along with the fundamental train frequency (FTF). It can be seen that all these frequencies are proportional to the rotating speed of the machine (number of revolutions per minute of the rotating shaft, RPM), through coefficients determined by the geometry of the rolling bearing, usually available in online databases.

The vibration signals are sensed by an accelerometer fixed to the stator of the machinery as close as possible to the roller bearing, therefore the amplitudes of the sensed transient vibrations depend on the distance between the impact locations and the sensor, which can be fixed as for outer race faults or variable in the other two cases. The received signal is therefore a transient train that can be amplitude modulated.

Summarizing, a widely accepted mathematical model for the resulting bearing failure vibration signal, if no sliding occurs, is the following [33]:

(1) t=1+KFsin2πfF∑iAC12 1−−1i+AC22 1−−1i+1e−t−iffault τ sin2πfrest−iffault ut−iffault

where *f_fault_* represents characteristic frequency related to the particular failure (i.e., *f_fault_* is equal to BPFI for inner race faults, to BPFO for outer race ones or to 2 BSF for ball faults), *f_res_* is the machine component resonance frequency, and *f_F_* and *K_F_* represent the amplitude modulation frequency and modulation index, respectively. Finally, *A_c_*_1_ and *A_c_*_2_ represent the amplitudes of the transient vibrations.

More in detail, for an outer race fault the defect is in a fixed position with respect to the sensor so *K_F_* = 0, *f_F_* = 0 and *A_c_*_1_ = *A_c_*_2_. For an inner race defect, the vibration signal is amplitude modulated since the defect moves with respect to the accelerometer, being integral with the rotating shaft. The modulation frequency *f_F_* is equal to the machine rotating frequency RPM/60, and the two amplitudes *A_c_*_1_ and *A_c_*_2_ are equal.

Finally, in the rolling element defect, the two amplitudes *A_c_*_1_ and *A_c_*_2_ are different, since they are associated with the defect hitting the inner race and the outer race alternately, during the spinning of the ball; in this case there is also the modulation effect given by the movement of the rolling element, integrally mounted in the cage. The modulating frequency is related to the cage speed and the frequency of the modulation is called fundamental train frequency (FTF). In this case, the failure frequency *f*_fault_ is equal to two times the characteristic frequency BSF.

Note that with the above-described model, the vibrations’ signals are always periodic.

**Figure 3:**
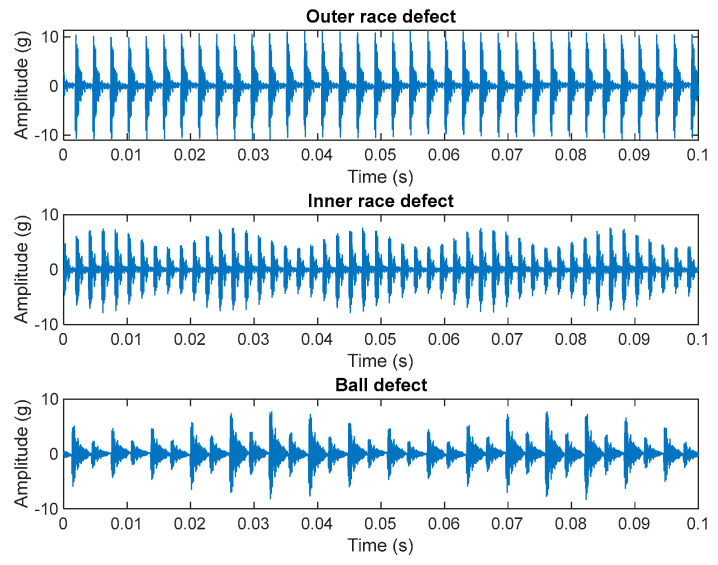
Simulated signals as per Equation (1), considering the same rolling bearing, for the three defect classes. In the reported example, fs = 10 kHz, RPM = 3000 rpm, BPFO = 361 Hz, BPFI = 488 Hz, BSF = 161 Hz, FTF = 21 Hz.

Considering the mathematical representation of the three different bearing failure classes, it is possible to distinguish the type of failure as well as the presence or absence of the failure, analyzing the signals either in time or in the frequency domain. Figure 3 represents simulated signals according to Equation (1) for the three failure classes mentioned above. The signals are obtained considering the characteristic frequencies of a typical rolling bearing. As can be noticed from the simulations it is possible to easily detect the differences between the three failure classes in terms of frequency and envelope.

Obviously, the pulse train nature of fault vibration signals is associated with wide band spectra, due to the high frequency content of the transients, while the features meaningful for diagnosis reside in the low frequency content.

Therefore, to acquire fault vibration signals, large band sensors and readout electronics and high sampling frequencies (*f_c_*) are needed together with long observation windows. Constraints on the large analog bandwidth and high sampling rates are posed by the transient frequency band, whereas the observation window length must be selected on the basis of the fault frequencies, which in turn change with the RPM and are usually less than 10 RPM. Many preprocessing techniques or classifier architectures need to be adapted, when changing the ratio *f_c_*/RPM, even if this problem is often disregarded in the literature [34,35,36].

### 2.2. Real Failure Behavior and the Measurement Problem

The mathematical representation of the bearing failure vibration signals in a series of amplitude modulated damped sinusoids, as in Equation (1), is a simplification of a complex scenario. In real cases, the behavior of the vibration signals is more complex. Apart from sliding, there are effects of attenuation in the signals given by the machine structure as well as related to the type of failure.

**Figure 4:**
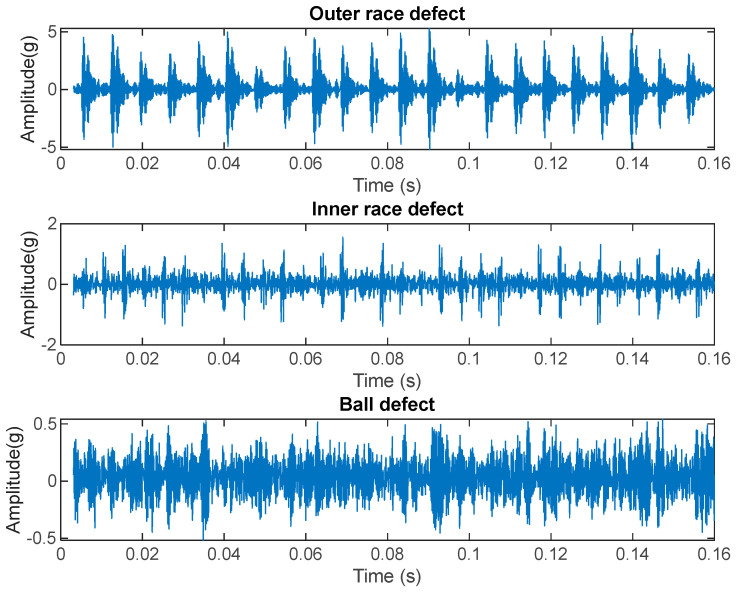
Real vibration signals from an online database (CRWU) for the three different failure classes; top: outer race, mid: inner race, bottom: ball. The signal to noise ratio decreases in inner race defect and the ball defect signals, due to the decreased amplitude of the vibrations, caused also by larger distance between the sensor and the defect.

Figure 4 represents real vibration signals extracted from an online database (the Case Western Reserve University database). As it can be noted, there are noticeable differences with respect to the emulated signals. Even if the outer race defect (on top) produces vibrations well described by Equation (1), for the other two classes, the theoretical behavior is barely visible. In particular, for rolling element failure, the amplitude of the vibration signal is quite low with respect to the other cases and the behavior of the failure in terms of damped vibrations is almost not perceivable.

This is given by the fact that especially for ball defects, the fault is present in a small part of the bearing and the impacts with the other bearing components create little amounts of energy with respect to the other failures. Moreover, the amount of energy created by a certain fault during the rotation of the shaft is also related to the amount of mechanical load and to the rotation speed of the shaft.

Besides the amount of energy generated by a fault impact, there is the aspect of the sensor position with respect to the impact location. In real case scenarios, for instance in harsh environmental conditions scenarios, such as in gas turbines, the vibration sensor placement is always a problem. For the best detection of the bearing vibration behavior, the sensor should be placed as near as possible to the bearing to be monitored or on the bearing itself; however, it is not always possible to place the sensor in the best position. High temperatures, mechanical constraints, and geometries usually determine placements far from the very bearing that must be monitored, increasing the distance between the sensor and the vibration source, which leads to the attenuation of the vibration signal and to the deteriorating of its information content.

A placement far from the bearing to be monitored could also cause other problems related to interferences from other vibration sources, for instances vibrations created by gear transmissions, chains, gearmotors and so on. In this context it is not possible to recover the characteristic behavior of the bearing failure.

It is important to also stress that the signal vibration behavior shown in Figure 4 is present only if no sliding occurs during the rotation of the bearing components. Sliding may arise in case of high mechanical loads; when it occurs the fundamental train frequencies vary, with respect to those reported in Section 2 related to the bearing geometry and dimensions, and the vibration signal loses its periodicity.

As previously stated, bearing failures progress gradually over time, often originating in a single bearing component like a race but then escalating to multiple components, leading to a more complex vibration signal with new harmonics which deviates from the conventional model. Moreover, in demanding environments, like high temperatures, suitable accelerometers might be lacking for wideband measurements. In this case, the accelerometer acts as a resonant low pass filter, heavily distorting the vibration signals.

**Figure 5:**
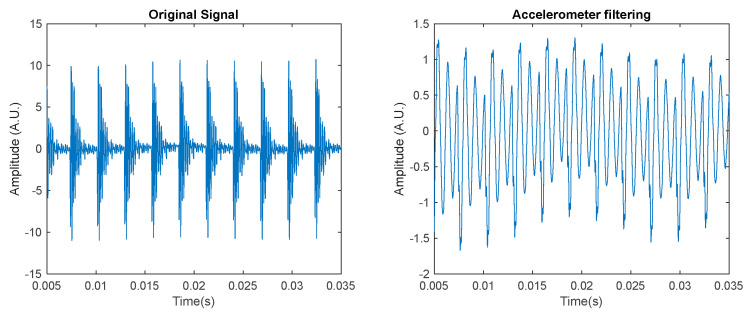
Example of accelerometer filtering. Left: vibration signal response, outer race, resonant frequency f_res_ = 7 kHz. Right: simulation of the sensor output considering the sensor to behave as a second order resonant system, with resonance at *f_n_* = 1 kHz and quality factor Q = 10.

Figure 5 represents an example of the output of an accelerometer whose bandwidth is much lower than the bandwidth of the signal to be measured. As it can be noticed, from the accelerometer output signal, the signal is heavily distorted, especially due to ringing.

The information that can be recovered from the signal in all these critical but realistic cases presented in this subsection, is only related to the vibration amplitude, and the sensor output signal can be exploited only for alarm, i.e., for fault detection, and not for fault location.

## 3. Failure Detection: Approach and Neural Network Aided Failure Classification

From a failure detection perspective, classifying the vibration signals according to the characteristics related to the mathematical representation of the three bearing failure classes is surely viable, as discussed above. Nevertheless, as discussed in Section 3.1, in real cases, the behavior related to the defect type is a characteristic of the signal which is not always present when a failure happens. For this reason, to implement a failure detection algorithm which is also valid in complex real scenarios (sliding, multiple faults, non-stationary machine regimes or fault conditions) it is important to focus on metrics which are based not only on the analysis of the vibration signal frequency content near the bearing fault characteristic frequencies but also on other metrics, based on other signal properties.

What is always true is that when a failure occurs there will be a change of the vibration signal behavior with respect to the one recorded in ‘normal’ conditions.

Typical approaches for the bearing early failure detection, implemented in condition monitoring setups, such as in oil and gas rigs and in wind turbines, for instance, rely on the analysis of the vibration signal during the machine operations, by evaluating the magnitude of its deviation from the ‘normal operation baseline’ exploiting several different metrics, e.g., in the time domain such as the root mean square value (rms) or the kurtosis over a time window lasting some rotation periods. An approach of this kind allows for detecting the presence of the failure but not for discriminating among the various types of failures.

**Figure 6:**
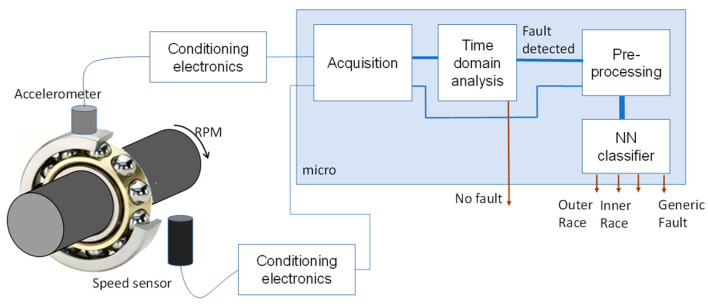
Diagnosis system block diagram. The system uses rotational speed and vibration data from an accelerometer. Faults are detected using conventional techniques; a neural network (NN) classifier is then employed for fault localization when a fault is identified.

Therefore, in this paper we propose the approach shown in Figure 6, which assumes the adoption of a classical technique to detect the presence of a fault, and in case of detection it exploits an artificial neural network-based classifier to locate the fault when possible. The classifier operates with preprocessed data, and the preprocessing technique is aimed at reducing as much as possible the dependence of the classifier on the machine working conditions such as the rotating speed, transient shape (i.e., machine resonant frequency and decay time), and the signal acquisition system set-up (sampling frequency).

Both the preprocessing and classifier are simple, low-computational burden techniques. They demand only a small amount of hardware resources, making them well-suited for deployment in low-complexity microcontrollers. Moreover, their design facilitates edge data processing in distributed sensor nodes.

### 3.1. Vibration Signal Preprocessing

The proposed feature extraction algorithm operates in the time domain and aims at transforming the time domain signal into an image, i.e., an arbitrarily long-time sequence into a fixed sized matrix.

However, processing the signals in the time domain has some obvious drawbacks. As the speed varies, considering the sampling frequency fixed, the vibration signal periods will be composed of a variable number of samples related to the ratio between the sampling frequency and the rotating speed. Several works exploit time-domain signals to realize 2D features of the different bearing failures, used as input of various type of classifiers [30,31,32]. In these cases, the 2D features are obtained by representing a window of the time signal having a fixed length using consecutive rows or columns of a matrix. However, in these works, the rotation speed is always considered constant. Regarding the 2D representation, considering a fixed sampling frequency will be influenced by the rotating speed.

To have a representation independent from the rotating speed, resampling the signal, with a sampling period related to the speed might be a feasible solution requiring some additional computational burden.

In this paper, by exploiting the a priori knowledge of the rotation speed, a 2D representation of the time-domain signal is proposed which is related only to the type of failure if present and if no sliding occurs. In particular, three *K* × *K* square matrices $F_{fault}$ (fault = O, I, B, where O indicates Outer Race Fault, I Inner Race Fault, whereas B indicates Ball defect) are built, with the following approach.

At first, for all the possible fault frequency *f_fault,_* defined in Section 2 (*f_fault_* = BPFO, BPFI, 2BSF), the three fault periods in terms of number of samples, *N_fault_,* are found as follows:

(2) $$N_{fault}=\lfloor \frac{f_{c}}{f_{fault}}\rfloor$$

Then, a time window of the sampled signal of length *T_w_* = *NT_c_* is considered, where $T_{c}=\frac{1}{f_{c}}$ is the sampling period, $f_{c}$ is the sampling frequency, and $N>\max(N_{fault})$, such that:

(3) $$s_{c}\left(n\right)=s\left(nT_{c}\right)\;\;\;\;\;\;\;\;\;\;n=0,\ldots N-1$$

Subsequently, the sampled signal is normalized as follows:

(4) $$s_{n}\left(n\right)=\frac{s_{c}\left(n\right)-\min s_{c}\left(n\right)}{\max s_{c}\left(n\right)-\min s_{c}\left(n\right)\;}$$

Finally, each $F_{fault}$ is initialized to the null matrix and evaluated as described by the pseudo code in Table 1.

Each matrix $F_{fault}$ represents a rasterized version of the plot of the normalized signal as a function of its delayed version, i.e., the X-Y representation of the signal with respect to the delayed one, being the delay ideally $T_{fault}=\frac{1}{f_{fault}}$ and with a persistence given by the chosen *T_w_* value. Gray scale images corresponding to the 2D $F_{fault}$ matrices are obtained by representing the $F_{fault}$ entry values with 8 bits. Notice that couples of samples ($s_{n}\left(n-N_{fault}\right),\;s_{n}\left(n\right)$) having both small values (below the threshold value in Table 1), are discarded and not used for the evaluation of $F_{fault}$. In particular, in this work, the threshold is chosen as K/10, i.e., 1/5 of the signal peak value. This allows for discarding the signal samples not belonging to the vibration transient peaks, for limiting the amplitude dynamics of the formed image, and for reducing the number of bits needed for coding.

Notice that the matrix size *K* can be arbitrarily chosen and represents the signal amplitude dynamics. A *K* value in the range (100, 200) is more than reasonable, given the typical signals to noise ratios for vibration signals.

In the presence of a specific fault (I or O or B), in ideal cases, when the vibration signals are described by Equation (1) and when the noise and the discretization errors both in amplitude and the time domain are negligible, the matrices $F_{fault}$ assume two characteristic shapes. The matrix $F_{fault}\;$ related to the present fault will be a diagonal matrix and the corresponding image is a −45° line, because it is obtained using the existent $T_{fault}$, while the other two images will have the shape of a cross because, on average, there is no synchronicity between the x-signal and the y-signal, so when $s_{n}\left(n-N_{fault}\right)$ assumes large values $s_{n}\left(n\right)$ will be small. On the other hand, if the fault vibration signal, due to any real-world effect can’t be described by a periodic signal at all, the images associated with all the $F_{fault}$ matrices will appear as circles.

Therefore, the simultaneous analysis of the three images $F_{fault}\;\mathrm{with}\;fault=O,\;I,\;B$ can easily lead to the location of the fault, allowing for a classification with four classes, trains of transient vibrations associated to outer, inner races or ball defects, or faults described by vibrations with no detectable periodicity.

**Figure 7:**
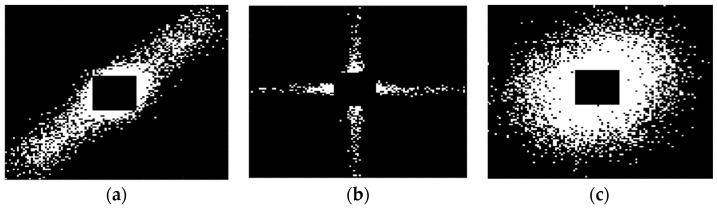
Representation of the images related to ***F****_fault_* matrices according to the type of signal and the delay used: (**a**) vibration signal is a train of transients, and the delay time is equal to the train period; (**b**) delay different from the train period; and (**c**) the vibration is not a transient train (Images are saturated for clarity’s sake).

Obviously in real cases the presence of noise and the sampling of the vibration signal causes these images to distort, obtaining features as those shown in Figure 7, where real signals were used to obtain the images. As can be noticed, it is still possible to distinguish if the delay is equal to the failure period or not from the proposed image, or if no periodicity is perceivable.

Finally, the three images derived from $F_{O,I,B}$ are concatenated to form the final ‘feature’ image used as an input to the following classifier, so the final fault feature, ***F***, is a K × 3 K image built as follows:

(5) $$F=\left[F_{O}\;F_{I}\;\;F_{B}\right]$$

**Figure 8:**
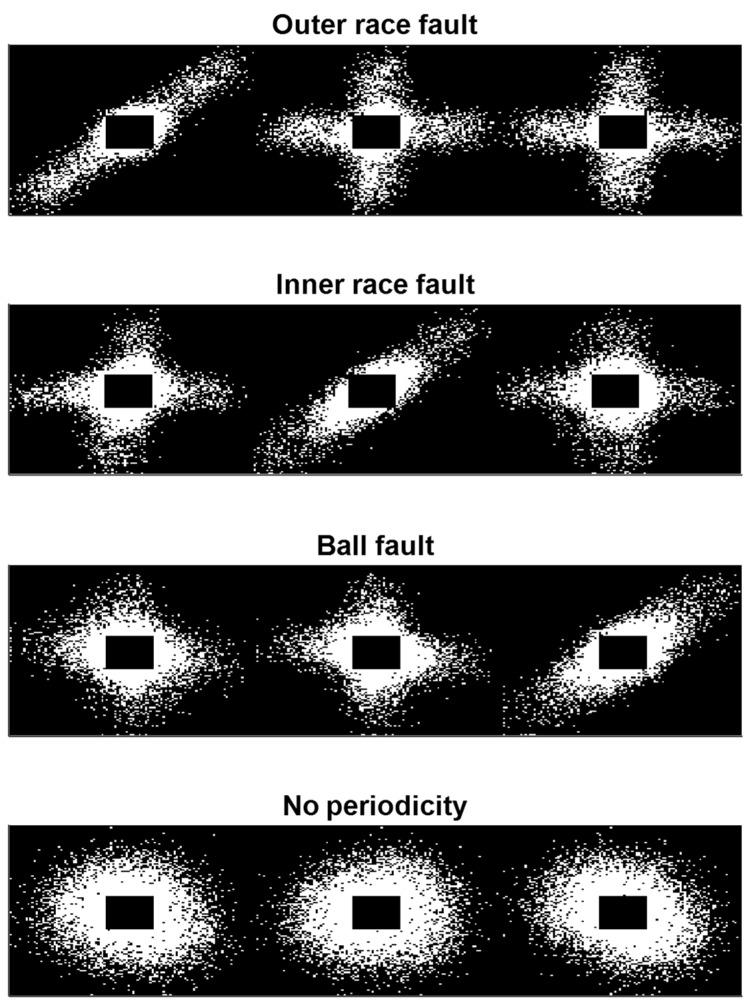
Example of fault feature images, ***F***, realized with the proposed method, using noisy signals (images are saturated for clarity’s sake). The example images were built exploiting signal windows made up of 6400 samples.

Figure 8 represents four examples of features images realized with the proposed method one for each different fault classes considered in this work, obtained with noisy real signals.

With this feature extraction procedure, the extracted features lose their dependency on the ratio between sampling frequency and machine rotating frequency, which is usually a scale factor in any other preprocessing and feature extraction technique either in the time or frequency domain. On the other hand, the type of roller bearing must be known a priori.

The pre-processing set up consists of selecting a threshold value (in Table 1) and the value of *N* (length of processed signal time window), This last parameter depends on the machine working conditions and can be adjusted based on the measured rotation speed as follows:

(6) $$N\geq \frac{n_{p}}{\min\left(f_{fault}\right)}f_{c}$$

thereby maintaining the same periods of *n_p_* equal, and also if the rotating speed varies.

Finally, and most importantly, the subsequent classifier design is independent from the machine working conditions, from the rolling bearing type, from the signal amplitude, from sampling time, and so on.

### 3.2. Neural Network Description

The used neural network (NN) was tailored to the realized images representing the bearing failures. A value *K* = 112 was selected for the ‘feature images’, therefore each fault feature image size is K × 3 K. In particular, the input layer was chosen according to the image size in pixels.

An average pooling layer was then implemented, which performs an average pooling of 112 × 112 with a stride of (112 112). The stride dimensions were selected according to the dimension of the $F_{fault}$ matrix dimensions. A 2D convolutional layer and a fully connected layer follow the average pooling layer. A softmax activated output layer is then used for the classification of the bearing failures. The output size of this latter is *k* = 4 for the classification of the three bearing failure classes (Outer, Inner, Ball) and for the class of faults associated with vibration signals characterized by no periodicity, which from now on is called ‘Noise’.

**Figure 9:**
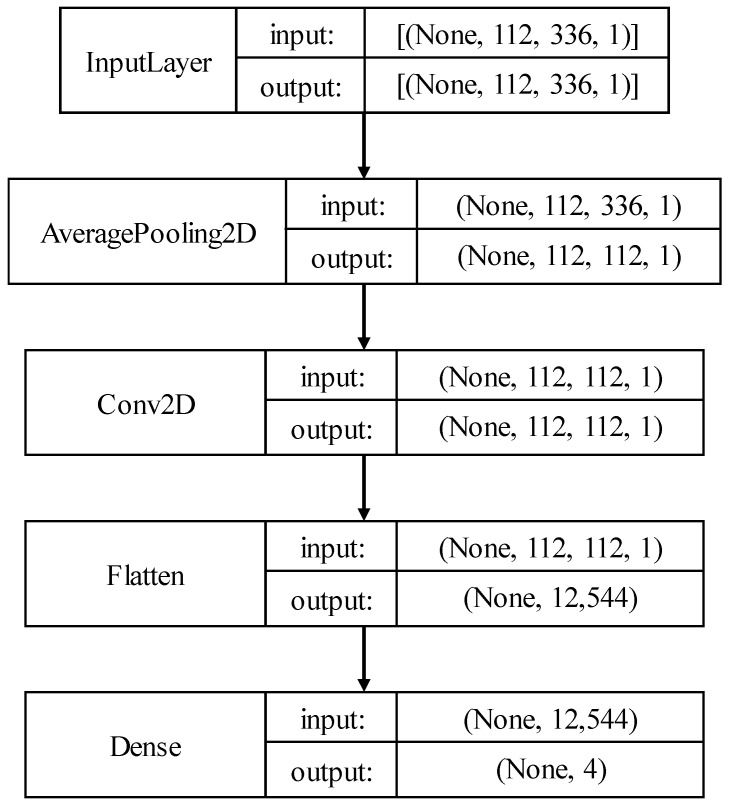
Neural Network Classifier block diagram. The classifier consists of five layers designed to match the characteristics of the input matrix. The complexity is intentionally low to minimize resource consumption, enabling deployment on resource-limited devices.

Figure 9 represents the NN architecture. The proposed network requires a memory capacity of 41 kB and can therefore be deployed in a wide range of low-cost, low resource devices such as microcontrollers or field programmable gate arrays (FPGA), even in the presence of hard memory constraints. Microcontrollers with limited random access memory (RAM), such as those in the STM32F4 family from ST Microelectronics, can be used for executing the network in local computing. Additionally, the resources needed for matrix image generation can be tailored to the microcontroller’s characteristics. The input sequence length can be chosen arbitrarily, as long as it covers a minimum number of periods of the lowest frequency defect in the acquired window.

Using an FPGA for the entire evaluation would entail an extra microcontroller for signal acquisition and matrix image generation. However, this setup could potentially reduce computation time.

Notice that the image processing task in this specific application is well defined because it consists of the recognition of few image types, with the need of rejecting the blurring effects of noise and of discretization (sampling). Therefore, available standard networks for image processing such as MobileNet and ResNet aimed at the solution of much more complex problems are not the best choice due to their complexity and to the operations such as rotations and striding performed to the image matrix (as shown later in this paper).

## 4. Datasets

Different datasets were exploited to train and test the proposed NN, based on emulated signals and on real signals available from online databases.

### 4.1. Emulated Signal Dataset

Different datasets were obtained using emulated signals generated with a test bench, previously developed by the authors [1] and based on an excitation shaker (B&K 4809). The shaker was driven by means of a power amplifier, whose input signal has been obtained mixing the outputs of two arbitrary waveform generators (AG33220A). Emulated signals, having the mathematical description of Equation (1), were obtained considering the three types of faults (I, O, B) for a real rolling bearing (type 6205-2RS JEM SKF) and for different rotation speeds up to 45 Hz.

The datasets were obtained considering different resonance frequencies, *f_s_*, up to 10 kHz.

The fourth class of faults, representing faults with no evident periodicity (the related class is named ‘Noise’), was emulated by driving the shaker with white Gaussian pseudorandom noise signals generated by a waveform generator (AG33220A) added to sinusoidal waveforms at a frequency equal RPM/60.

Three different types of accelerometers were fixed to the shaker and used to sense the emulated fault vibration signatures. In particular, the used sensors were a piezoelectric accelerometer (Bruel & Kijer 4326) having a 10% bandwidth of 16 kHz and two embedded triaxial devices based on MEMS devices (Analog Devices ADXL1005), previously realized and characterized in [33,37,38] having 10% bandwidths of 12 kHz and 14 kHz, respectively. The vibrations’ signals were acquired by means of a 16-bit DAQ board, with different sampling frequencies up to 160 kHz. The window length *N* used to form the fault feature images (image size 112 × 336) was chosen as per Equation (6) with *n_p_* = 8.

The training and validation dataset was obtained by preprocessing emulated signals relative to the four fault classes: ‘Noise’, and ‘O = Outer race Fault’, I = Inner Race fault’ and B = Ball defect’, by considering a machine resonant frequency of 10 kHz, and RPM = 1800 rpm, so that, BSF = 70.7 Hz, FTF = 11.9 Hz, BPFI = 162.5 Hz and BPFO = 107.5 Hz (see also Table 2).

All signals used to build the training and validation dataset were sampled at different sampling frequencies of 26 kHz, 53 kHz and 160 kHz.

The training and validation dataset contains 588 images, 147 per class.

Test datasets were obtained by injecting noise with different magnitudes, by varying the machine rotation speed, the sampling frequency, as well as the machine resonant frequency (values used: 7 kHz, 8 kHz and 10 kHz). These datasets were approximatively the same size of the training dataset with evenly distributed examples per class.

In particular, noisy signals, *s_cn_*(*n*) used to form the test datasets were obtained exploiting the following relationship:

(7) $$s_{cn}\left(n\right)=s_{c}\left(n\right)+n_{g}\left(n\right)+n_{RPM}\left(n\right)$$

where *n*, $n_{g}\left(n\right)$ is a gaussian white noise, whereas:

(8) $$n_{RPM}\left(n\right)=\overset{5}{\underset{m=1}{\sum }}a_{m}\sin(\frac{2\pi mRPM}{60}nT_{c}+\phi_{m})$$

represents the vibration related to the machine rotation.

**Figure 10:**
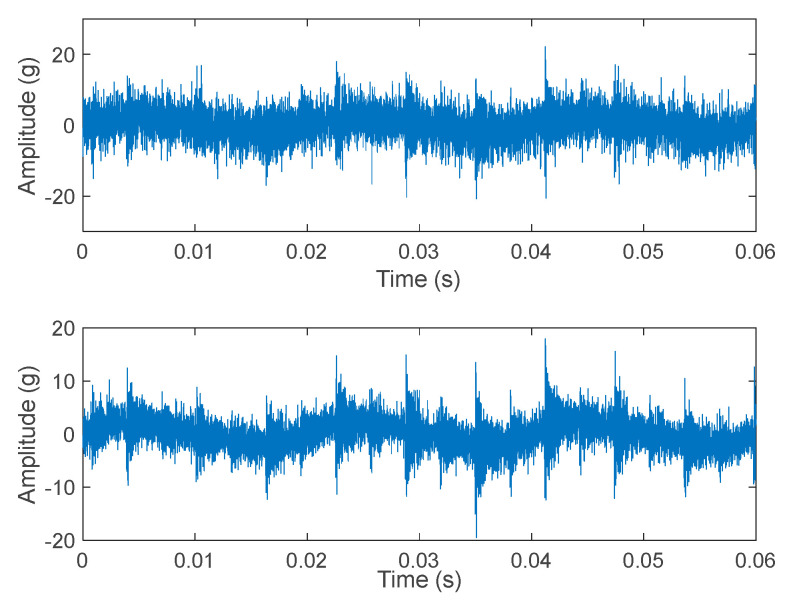
Example of emulated noisy signals. Ball fault, machine vibration at 10 kHz. Above: Gaussian noise standard deviation = 20% of the acceleration peak value, *a*_1_ = 10% of the signal peak value. Below: Gaussian noise standard deviation = 10% of the acceleration peak value, *a*_1_ = 10% of the signal peak value.

Standard deviations of the Gaussian noise with values up to 20% of the typical peak value of the vibration signals were used, so as for $a_{1}$, while $a_{m}$, for *m* > 1, were assumed fixed fractions of $a_{1}$. Finally, $\phi_{m}$ were random phases. Examples of noisy data sets obtained according to Equations (7) and (8) are shown in Figure 10.

### 4.2. Real World SIGNAL Dataset

The proposed classifier was further tested with real data available in the online database of the Case Western Reserve University (CWRU).

The signals available in this database are characterized by different rotation speed, sampling time, component vibration, natural frequency, and bearing geometry with respect to those used for signal emulation. The CWRU dataset is a popular, open-source, and easily accessible dataset. The CWRU bearing dataset is often used as a reference [39] dataset to test the performance of different ML and DL algorithms.

The bearing test rig arrangement used to obtain the CWRU data consists of a 2 hp Reliance electric induction motor, a torque transducer, a dynamometer, and control electronics. Two test bearings (fan end and drive end) support the motor shaft. Torque is applied to the shaft through a dynamometer and electronic control system. Some single point faults were induced in the rolling elements, the inner race and outer race, and each faulty bearing was reinstalled on the test rig. SKF (and equivalent NTN) bearings were used. Acceleration signals were measured at locations near to and far-off the motor bearings, by multiple sensors. Accelerometers were attached to the housing with magnetic bases. For the drive-end bearing experiments, signals were sampled at 12 kHz and 48 kHz, while fan-end signals were sampled at 12 kHz.

Drive end (DE) data were considered for the proposed system test. A dataset of 387 images, 129 for each class was used to test the NN network. The dataset was created with only three classes, corresponding to the three failures documented in the database (I, O, and B faults). This differs from the datasets generated using emulated signals (outlined in Section 4.1), which featured four classes, including samples representing faults without noticeable periodicity (Noise). The RPM was 25 Hz and the coefficients needed to obtain the different faults are reported in Table 2.

**Figure 11:**
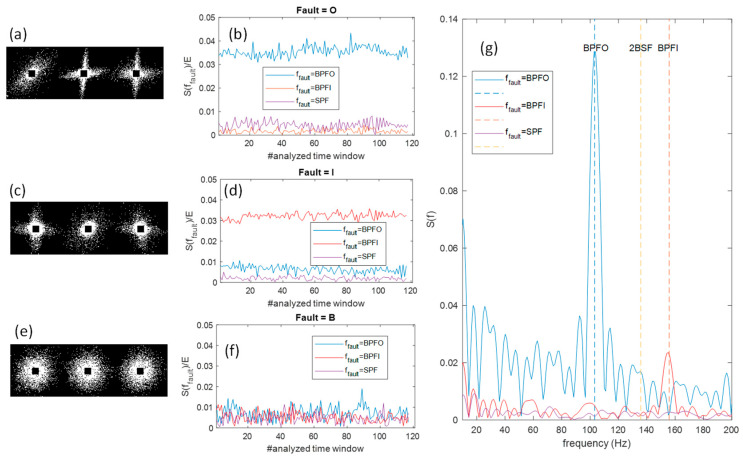
Results of different analysis types on data from the CRWU database. Analysis of time window of signals in the ‘O’ fault class: (**a**) feature image obtained with the preprocessing proposed in this paper; (**b**) amplitude of the spectral components (of the signal envelope) located at BPFO, BPFI and 2BFS normalized with respect to the spectral energy in the base-band (20 Hz–2 kHz). Analysis of signals form the ‘I’ fault; (**c**) feature image obtained with the preprocessing proposed in this paper; (**d**) amplitude of the spectral components located at BPFO, BPFI and 2BFS normalized with respect to the spectral energy in the base-band (20 Hz–2 kHz). Analysis of signals form the ‘B’ fault; (**e**) feature image obtained with the preprocessing proposed in this paper; (**f**) amplitude of the spectral components located at BPFO, BPFI and 2BFS normalized with respect to the spectral energy in the base-band (20 Hz–2 kHz); (**g**) envelope spectra obtained from three signals classified as ‘O’, ‘I’ and ‘B’ faults, respectively.

The signals contained in the database and considered for the dataset construction were also analyzed with a traditional technique based on zero-padded DFT performed on the signal envelope and using signal windows with a length selected according to Equation (6) and n_p_ = 8. The plots in Figure 11b,d,f report the result of this analysis on signals of the three different fault types (Figure 11b for ‘O’, (d) for I and (f) for ‘B’) and for all the time windows used to build the database. In particular, Figure 11b,d,f, show the amplitude of the spectral components located at BPFO, BPFI, and 2BFS normalized with respect to the spectral energy in the base-band (20 Hz–2 kHz).

In the presence of the expected characteristic fault periodicity one of these spectral components has a value larger than others indicating the presence of a large spectral peak, whereas in its absence no large peak at the expected fault frequency is found and all the three normalized components are similarly small. It can be seen that in the case of O and I fault, the periodicity is present, whereas all the B faults in the database lack this feature (see also the time-domain examples in Figure 4). This was also confirmed by an STFT analysis using 10% overlapped windows, which evidenced the absence of persistent spectral peaks. Therefore, we effectively deem that the actual class of the B defects in the CRWU database should be ‘Noise’, and as such we expect an almost full misclassification of the B class, which will be recognized by the trained network as ‘Noise’. The behavior observed for the ‘B’ fault in this database can be explained by the many non-idealities discussed in Section 2, which can be more severe in case of ball defects.

In Figure 11g three envelope spectra obtained from three signals classified as ‘O’, ‘I’ and ‘B’ faults are shown as examples of the typical behavior.

Finally, some examples of feature images obtained as signals from the CRWU data base are shown as the leftmost plots of Figure 11a for an ‘O’ fault, (c) for an ‘I fault, whereas (e) for a ‘B/Noise’ fault), to underline how, as expected, the proposed preprocessing technique generates feature images with the same characteristics as those obtained with the emulated signals independently on the signal characteristics (sampling rate, rotation speed, vibration transient shape etc.).

## 5. Experimental Results

### 5.1. Training and Validation Results

Training and validation were performed exploiting an emulated signal dataset, as described in Section 4.1, with a total of 588 elements, 147 per class.

**Figure 12:**
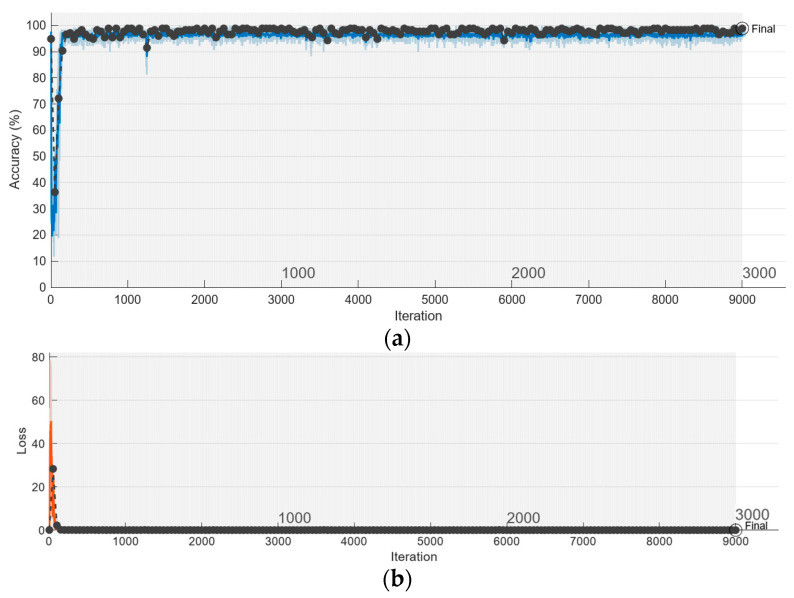
Training and validation results, in terms of accuracy and loss. Training was stopped after 3000 epochs: (**a**) final training and validation accuracy were 99.45% and 98.86%, respectively; (**b**) training and validation loss.

Figure 12a,b represent the results obtained during model training in terms of training and validation accuracy as well as loss. The model training was stopped after 3000 epochs and reached the remarkable training and validation accuracies of 99.45% and 98.86%, respectively.

### 5.2. Test Results

The trained network was tested with the test datasets described in the previous sections, obtained from emulated data having different characteristics with respect to the training data as well as with data coming from the CWRU database.

#### 5.2.1. Test with Emulated Data

The results obtained with the test dataset described in Section 4.1 are summarized in Table 3.

As can be noted, five different scenarios were represented by the datasets used for the experiments whose results are reported in Table 3. Regarding the dimensions of the dataset reported in Table 3, the values correspond to the total dataset size. The samples were evenly distributed across the four classes (O, I, B, Noise) for each dataset.

The results are excellent. In fact, the overall accuracy in all the scenarios is higher than 97%, pointing out the ability of the proposed diagnosis system to generalize, showing performance almost independent from the machine rotation speed, the sampling frequency, and the resonance frequency considered. Moreover, the robustness against noise is remarkable.

**Figure 13:**
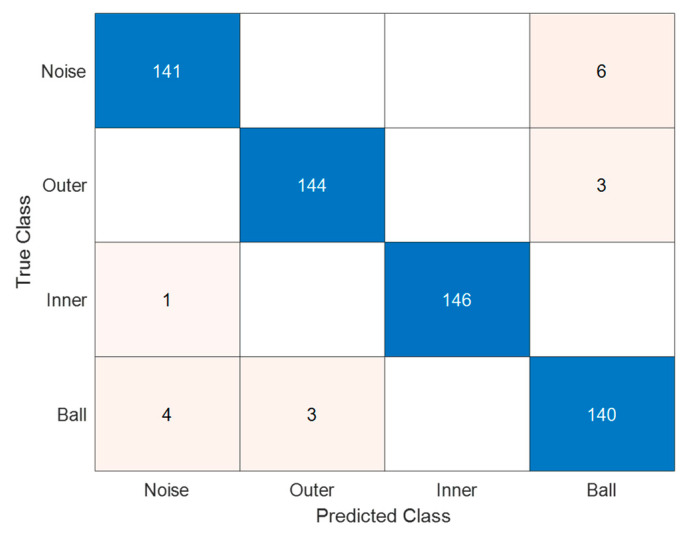
ML model test confusion matrix, signals with increased noise. Each class contains 147 samples; misclassification occurs mostly in the discrimination of ball defect and noise.

As an example, Figure 13 represents the machine learning (ML) model confusion matrix with the dataset number 1 (as per Table 3), characterized by the maximum noise level. The dataset contains 147 samples for each class, with a total number of samples equal to 588. It can be noticed that the less critical recognition is the one related to inner race faults.

Furthermore, the network’s misclassifications primarily involve mistaking noise for ball faults and vice versa. This observation is supported by Figure 8, which illustrates that with an equal level of injected noise, O and I faults produce images that are more distinguishable from noise compared to B defects.

#### 5.2.2. Real Signals from CWRU

**Figure 14:**
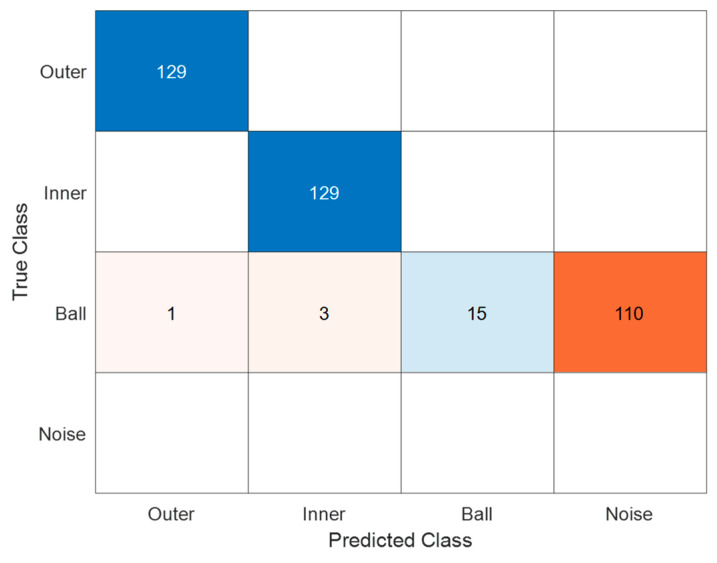
ML model test confusion matrix, signals from CWRU database. Each class has 129 elements. The dataset includes only I, O, and B classes due to the absence of Noise class in the database. The high misclassification in the B class is attributed to the lack of periodicity in the real signals, preventing accurate classification.

In Figure 14, the confusion matrix of the Machine Learning (ML) model is presented, utilizing signals from the online database CWRU. The dataset is realized as described in Section 4.2, containing a total of 387 elements equally divided in the three classes (I, O and B) present in the CWRU. As anticipated, a significant portion of signals corresponding to ‘B’ (ball) defects from CWRU (Case Western Reserve University dataset) has been classified into the ‘Noise’ class. This outcome was expected and was discussed in the previous section. The reason behind this classification is that no discernible periodicity in the vibration signals could be detected, even when employing different and alternative analysis methods.

Consequently, these signals can appropriately be assigned to the ‘Noise’ class, as the fault diagnosis system’s design relies on the presence of a typical periodicity for categorizing signals into the ‘O’, ‘I’, and ‘B’ classes. On the other hand, any vibration signal exhibiting a sufficiently large amplitude to indicate faulty and irregular bearing behavior, but lacking periodicity is classified into the ‘Noise’ class, since similar signals are not typical of ‘B’ faults, but they can be originated by any other fault e.g., in the presence of sliding, in non-stationary conditions where the faults change and evolve during measurement and so on.

The results obtained with this dataset, which concerns a different measurement set-up, a different roller bearing, and different machine working conditions confirm the ability of generalizing of the proposed approach.

### 5.3. Comparison with Standard Pretrained and Prebuilt NNs

The outcomes achieved through the proposed network architecture were compared with the performance of two prebuilt NNs: MobileNet V2 and ResNet 50. These prebuilt networks were adapted from the Matlab Deep Network Design toolbox, involving modifications to the first two input layers (image input layer and first convolutional layer) to match the dimensions of the images created for different datasets. Adjustments were also made to the output layers of both prebuilt NNs to align with the required number of classes for the classification task.

Both prebuilt NNs underwent training and validation using the same dataset employed for the proposed network, achieving 100% accuracy in both training and validation. Testing of the trained networks used the datasets applied to test the proposed NN. Table 4 provides a summary of the three NNs, comparing resource requirements and testing accuracy results across three distinct databases.

The comparison reveals that the proposed approach demands fewer resources than standard prebuilt NNs designed for image classification. Regarding testing accuracies across three distinct datasets unrelated to the training dataset, it is evident that the proposed network achieves superior accuracy compared to the two prebuilt NNs, despite their high training accuracy.

As discussed in Section 3.2, this outcome arises from the typical image classification NNs, like the two utilized here, containing intermediate layers that result in the loss of spatial information. Given that the images portraying different failures retain well-defined spatial information, it is imperative to preserve the image proportions even within the intermediate layers of the network, which the proposed approach accomplishes.

## 6. Conclusions

In this study, we have introduced a machine learning methodology for diagnosing rolling bearing faults through vibration signal analysis. The proposed approach encompasses a lightweight preprocessing technique that leverages machine rotation frequency measurements. This enables the extraction of fault features without reliance on machine operational conditions, acquisition sampling frequency, or the responses of mechanical components and front-end electronics. The subsequent fault classification process is facilitated by a lightweight neural network (NN), facilitating the implementation of the entire diagnostic system using low-complexity, cost-effective hardware components. This design is tailored for embedded systems development.

The effectiveness of our developed diagnostic system was validated using a sizable dataset created from diverse emulated data, each representing distinct characteristics. Subsequent testing included scenarios with varying rotation speeds of the shaft. Across all tested scenarios, the classification accuracy consistently exceeded 97%, even in the presence of substantial noise (with root mean square values up to 20% of the signal peak value).

Furthermore, our system performance was assessed using real-world data sourced from the CRWU database, demonstrating its capacity for generalization. The novelty of our approach, built upon classical fault diagnosis principles, is primarily attributed to the novel structure of the preprocessing algorithm and the generation of fault signature images. These images are solely dependent on the inherent periodicity intrinsic to the fault vibration signal. Importantly, they eliminate dependency on rotation speed, acquisition sampling rate, and the specific transient vibration responses of mechanical components.

This transformative aspect simplifies the classification task, permitting the use of straightforward NN architectures. Moreover, this innovation facilitates training the network on a reference working condition database or its emulation, subsequently deploying the trained network in varying operational conditions. In essence, our methodology capitalizes on the intrinsic characteristics of fault vibration signals to create a versatile and efficient diagnostic system that can be confidently implemented across diverse settings.

## Figures and Tables

**Table 1:** Matrix $F_{fault}$ derivation, O stands for Outer Race Fault, I stands for Inner race fault, B stands for Ball fault.

$\mathrm{for}\;\mathrm{fault}=O,I,B\;\left(f_{\mathrm{fault}}=\mathrm{BPFO},\mathrm{BPFI},2\mathrm{BSF}\right)$
$N_{\mathrm{fault}}=\frac{f_{c}}{f_{\mathrm{fault}}}$
$F_{\mathrm{fault}}=0$
$\mathrm{for}\;n=N_{\mathrm{fault}},\ldots ,N-1$
$\mathrm{if}\;s_{n}\left(n-N_{\mathrm{fault}}\right)\;and\;s_{n}\left(n\right)>threshold$
$i=\lfloor s_{n}\left(n-N_{\mathrm{fault}}\right)K\rfloor$
$j=\lfloor s_{n}\left(n\right)K\rfloor$
$F_{\mathrm{fault}}\left(i,j\right)=F_{\mathrm{fault}}\left(i,j\right)+1;$
End
*End*
*End*

**Table 2:** $f_{defect}$ coefficients for the bearings used in this paper.

Bearing Type	Multiple of Shaft Speed			
	BPFI	BPFO	FTF	BSF
6205-2RS JEM SKF (DE)	5.415	3.585	0.3983	2.357

**Table 3:** Tests with Emulated Signals Datasets—Accuracy Results.

Dataset Number	Sampling Frequency	RPM	Noise	Dataset Dimension	Overall Accuracy
1	53 kHz	1800	20%	588	97.11%
2	26 kHz	1800	10%	252	96.83%
3	160 kHz	1800	10%	714	97.65%
4	53 kHz	900	10%	152	98.68%
5	53 kHz	2700	10%	320	98.75%

**Table 4:** Characteristics comparison of the proposed NN with prebuilt NNs, in terms of resources and test accuracies results.

NN Name	Network Weight	Layers	Accuracy (CWRU)	Accuracy (Emulated, 26 kHz, 1800 RPM, 10% Noise)	Accuracy (Emulated, 53 kHz, 1800 RPM, 20% Noise)
Proposed	41 kB	5	70.51%	96.83%	97.11%
MobileNetV2	8.37 MB	154	35.43%	77.78%	60.31%
ResNet50	85.69 MB	177	35.66%	80.25%	57.19%

## Data Availability

No new data were created or analyzed in this study. Data sharing is not applicable to this article.
